# Context Matters: Teaching Styles and Basic Psychological Needs Predicting Flourishing and Perfectionism in University Music Students

**DOI:** 10.3389/fpsyg.2021.623312

**Published:** 2021-03-02

**Authors:** Dora Herrera, Lennia Matos, Rafael Gargurevich, Benjamín Lira, Rafael Valenzuela

**Affiliations:** ^1^Department of Psychology, Pontifical Catholic University of Peru, Lima, Peru; ^2^Department of Social Psychology and Quantitative Psychology, University of Barcelona, Barcelona, Spain

**Keywords:** perceived autonomy support teaching style, perceived controlling teaching style, basic psychological need satisfaction, basic psychological need frustration, perfectionism, flourishing

## Abstract

Professional musicians are expected to perform at a very high level of proficiency. Many times, this high standard is associated with perfectionism, which has been shown to prompt both adaptive and maladaptive motivational dynamics and outcomes among music students. The question about how perfectionism interplays with motivational dynamics in music students is still unanswered and research within this line is scarce, especially in Latin America. In the light of Self-Determination Theory (SDT), this cross-sectional study investigated the relationship between the perceptions of motivational context (teachers' motivating styles: autonomy supportive or controlling), basic psychological needs (satisfaction/frustration), perfectionism (adaptive/maladaptive), and flourishing in University music students from Lima, Peru (*N* = 149; mean age = 20.68, *SD* = 3.03; 71% men). We performed a path analysis testing a model in which motivational teaching styles predicted both, perfectionism and flourishing via need satisfaction and frustration. The model's fit indices were ideal [χ^2^ (7, *N* = 143) = 7.48, *p* = 0.300, CFI = 0.998, TLI = 0.992, RMSEA = 0.021, SRMR =0.040]. In this model, perceived autonomy supportive style predicted need satisfaction positively and need frustration negatively; perceived controlling teaching style did not predict need satisfaction nor frustration. In turn, need satisfaction positively predicted adaptive perfectionism (i.e., high standards) and flourishing; whereas, need frustration predicted maladaptive perfectionism (i.e., discrepancy). These results shed light on the relevance of perfectionism in the psychology of higher music education students. Lastly, we highlight the importance of autonomy support in fostering adaptive high standards and flourishing in music learning.

## Introduction

Life without music would be unimaginable. No culture has existed without it (Brown et al., 2000). Musicality has been an integral part of human activity far earlier than first thought (Miller, 2009), and music-making is at least as old as humanity itself. Music is highly relevant, even for non-musicians, and it has evolved with the advent of language, figurative art, and human civilization (Higham et al., 2013).

Mastering musical skills requires experience and technical know-how that is usually acquired through formal instruction enabling high levels of performance (Bennett and Stanberg, 2008; Gaunt, 2011; Evans and Bonneville-Roussy, 2016). Consistent with these high expectations, evidence shows that perfectionism is regularly present among students of higher music education. Partly, because—as professionals—they will be held to a proficient performance standard (Grey, 2017).

Perfectionistic tendencies start manifesting early in students' lives, with prevalence increasing between the ages of 10 and 17, especially among girls (Patston and Osborne, 2016). Previous research has also stated that music learners' experiences may be influenced by different social contexts, like the family, from an early age (9–11 years old), given that the development of musical attitudes, intentions, and abilities can be explained by family background (Valenzuela and Codina, 2014).

Contextual influences are part of higher music education (i.e., University level). Students as pupils have to navigate the process of learning to perform their musical instruments at a proficient level through one-on-one tuition. At the same time, they deal with competitive social environments and face high levels of career uncertainty to be a successful musician. It is evident that music students have to deal with many pressures, and it is unavoidable that higher music education students will have to cope with these high-performance standards and challenges (Bennett and Stanberg, 2008; Gaunt, 2011; Evans and Bonneville-Roussy, 2016).

It is also important to consider the influences of context on motivational dynamics in higher music education. A theoretical perspective that pays attention to contextual influences is Self-Determination Theory (SDT). The theory underscores that some contexts may support the satisfaction of the three basic psychological needs, which are considered universal psychological nutrients to grow and develop (Ryan and Deci, 2000, 2017). These needs are (i) autonomy, (ii) competence, and (iii) relatedness (or non-isolation) (Deci and Ryan, 1985; Ryan and Deci, 2000, 2017).

Autonomy refers to the volition and experience of freedom when carrying out an activity; competence includes the feeling of effectiveness and confidence in pursuing a desired outcome; and relatedness implicates the feeling of being accepted by others and connected to others in a secure environment (Ryan, 1995; Ulstad et al., 2016; Ryan and Deci, 2017). These needs are preconditions for human growth and development (Ryan and Deci, 2017). In this regard, in music educational settings, an autonomy-supportive teaching motivational style can support students' basic psychological need satisfaction (Freer and Evans, 2019).

Considering the role that context can have in need satisfaction, research developed based on the SDT framework signals that teaching styles are crucial for students' motivation and wellbeing (Ryan and Deci, 2017). Thus, an autonomy-supportive context positively predicts basic psychological need satisfaction, (Weinstein and Ryan, 2010; Ryan and Deci, 2017); whereas the experience of need frustration occurs when basic psychological needs are thwarted by a controlling teaching style (Vansteenkiste and Ryan, 2013).

Autonomy supportive teachers play a relevant role in developing students' motivational resources. Such professors generally listen to pupils' perceived problems, respond to questions, offer alternatives to choose from, minimize external control, recognize and respect students' feelings and, lastly, also facilitate the use of specific learning strategies (Ulstad et al., 2016, 2018). Conversely, controlling teachers use the opposite style during instruction (Deci and Ryan, 1985; Ryan and Deci, 2017). They pressure students to think, feel, or behave in specific ways (Reeve et al., 2004; Assor et al., 2005; Reeve, 2009). Music students at the University level are generally faced with low levels of context-derived autonomy support, given that their professors tend to be prescriptive and this leaves little margin for students' input regarding practice activities, which in turn has effects on their experiences and motivation (Evans, 2015).

Need satisfaction matters in specific ways in arts and music education. High schoolers with higher levels of need satisfaction ascribed a higher value to instrumental practice, which in turn predicts their intentions of choosing elective music classes (Freer and Evans, 2017). There are also findings, in the field of arts, showing that basic psychological need satisfaction supports creativity, while basic psychological need thwarting is related to perfectionism in young ballet students (Nordin-Bates and Kuylser, 2020).

On the other hand, basic psychological need thwarting is associated with maladaptive perfectionism as well (Costa et al., 2015). It should be noted that, besides the importance of context for basic psychological need satisfaction, the satisfaction of these needs has been shown to predict flourishing; especially, relatedness need satisfaction has shown predominance in the prediction of flourishing among University students (Mesurado et al., 2016). And also participants of different generations and a wide range of ages have reported enjoyment for being involved in music learning programs in Australia (Ellis, 2018).

In the same vein, but from a person-context interaction perspective, individuals' immediate environments, including parental practices, family environment, and school type, may shape the development of perfectionism at a personal level (Flett et al., 2002; Hibbard and Davies, 2011; Hewitt et al., 2017; Curran and Hill, 2019). In the musical field, pupils with higher levels of perfectionism are prone to performance anxiety and to being concerned about making mistakes when performing. Interestingly, higher levels of perfectionism have been found among musicians with more years of experience, and the relationship between perfectionism and music performance anxiety increases during early adolescence until the end of schooling (Patston and Osborne, 2016). This particular association is very common among music students, as it has been reported in different ages and levels of ability and experience of music learners, including those enrolled in higher education (Patston, 2014).

Despite these negative associations, perfectionism also has a favorable dimension, associated with favorable outcomes at a psychological level. In this sense, Slaney et al. (2001) distinguished two different dimensions of perfectionism. The first denotes a high standard or striving-for-perfection, which has mainly adaptive aspects (Rice et al., 2014); and the other refers to self-discrepancy, which involves the difference between the person's real performance and their stated standard (Slaney et al., 2001) which is primarily maladaptive (e.g., Stoeber and Otto, 2006). These two dimensions of perfectionism are conceptually distinct and statistically independent and, thus, incompatible with a single bipolar dimension (Rice et al., 2014).

Research findings show that people who hold high personal standards but are not characterized by negative self-evaluation have been classified as normal, healthy, or adaptive perfectionists (Rice and Mirzadeh, 2000; McArdle and Duda, 2004). It has been also pointed out that a high standard of perfectionism is associated with positive results such as higher performance, academic achievement, self-confidence, and lower anxiety (Stoeber and Otto, 2006; Kira et al., 2018). In contrast, people who report high personal standards and strong negative self-evaluation tendencies, have been considered neurotic, dysfunctional, or maladaptive perfectionists (McArdle and Duda, 2004). When individuals perceive self-discrepancy between their high standards and their behavior, they tend to feel persistent self-blame, to self-evaluate in an overly critical manner, and to become concerned about others' criticism (Dunkley et al., 2003; Litz et al., 2009; Kira et al., 2018). In the same line, other findings have shown that higher discrepancy scores are significantly and negatively related to academic achievement. This self-discrepancy is maladaptive, as it led to difficulties in managing painful feelings and to a tendency to suppress them. And most worryingly, students who were characterized as maladaptive perfectionists reported higher levels of depression (Rice et al., 2014).

As previously mentioned, perfectionism is relevant among music students, and, thus, it comes to no surprise that contextual influences, such as controlling teaching style have been shown to lead to maladaptive perfectionism, low self-esteem, obsessiveness, anxiety, negative affect, and exhaustion (Haraldsen et al., 2020). This signals that maladaptive perfectionism may be a by-product of contextual influences, such as controlling teaching. Previous studies have also reported that University music students, when their basic psychological needs were satisfied by their learning contexts, preferred more challenging tasks, greater frequency of practice, and invested themselves in better quality practice as well (Evans and Bonneville-Roussy, 2016).

The relevance of understanding perfectionism and flourishing in students of higher music education is evidenced by the important downstream effects these two phenomena have on musicians' careers. However, these variables need to be analyzed alongside basic psychological need satisfaction/frustration, and considering context that supports or thwarts these needs, the satisfaction of which will ultimately predict students' wellbeing (i.e., flourishing) and adaptive perfectionism. This is particularly important for music students who are immersed in a competitive context and individual teaching.

**Figure 1:**
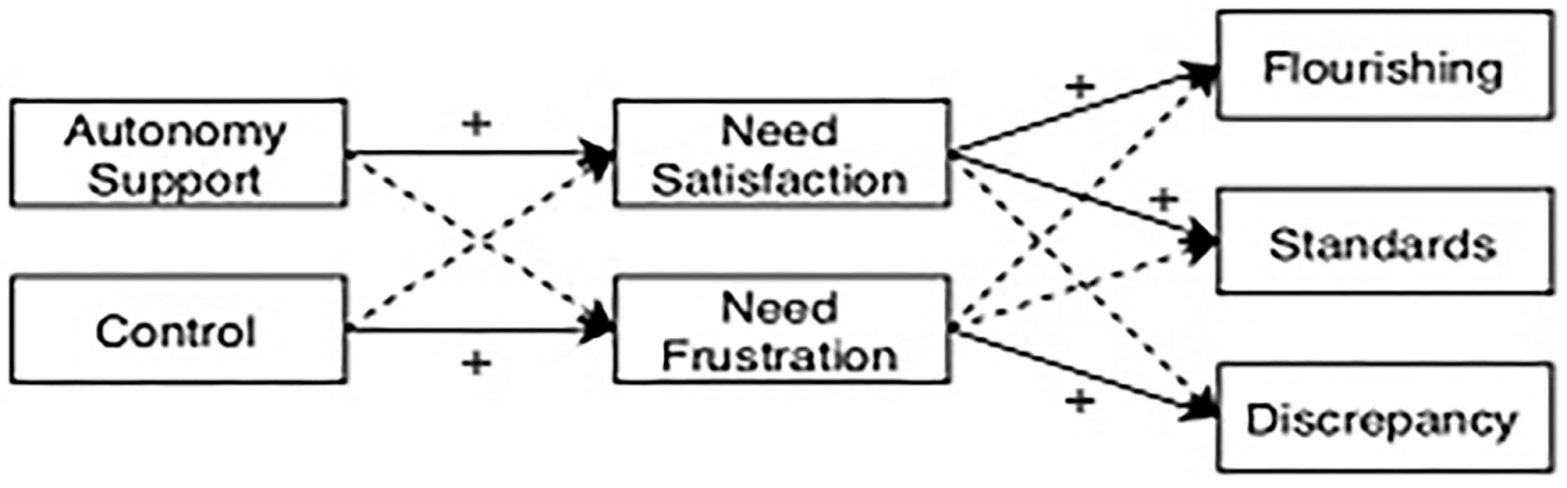
Hypothetical model.

Using SDT as a framework, this cross-sectional study aims to investigate the relationship between the perception of motivational context (teaching motivating style: autonomy support and control), basic psychological needs (satisfaction and frustration), perfectionism (standards and discrepancy), and flourishing in University music students. The hypothetical model postulates that a perceived autonomy-supportive teaching motivational style positively predicts adaptive perfectionism (standards) and flourishing, mediated by basic psychological need satisfaction; and that a perceived controlling teaching motivational style positively predicts maladaptive perfectionism (discrepancy) and negatively flourishing, mediated by basic psychological need frustration (see Figure 1). This goes in line with the bright and dark side of motivation respectively (Haerens et al., 2015)

## Methods

### Participants and Procedure

Participants were 149 undergraduate University music students from Lima, Peru. They were mostly men (71%, *n* = 106) and their ages ranged from 17 to 33 (*M* = 20.68, *SD* = 3.03) years old. The sample included students from first year (28.2%), second year (20.1%), third year (21.5%), or higher years (29.5%) in a 5-year program. Most of their parents had post-secondary education (97%).

The Ethics Committee of our University approved the research project. Participants signed informed consent and assent forms, which described the purpose of the study, and that their participation was voluntary and anonymous.

Assessments were conducted during regular class hours. After contacting University authorities, they provided classrooms and scheduled time for the students to fill-out the questionnaires, which took 20 min.

### Measures

All items were answered using a seven-point Likert-type scale ranging from 1 (totally disagree) to 7 (totally agree). All questionnaires, originally developed in English, were translated into Spanish in previous studies and were adapted for Peruvian University students of music for this study.

#### Teaching Motivational Styles

Perceived autonomy support from the teacher was measured using the six-items from the short version of the Learning Climate Questionnaire (LCQ—Williams and Deci, 1996) adapted for Peru (Matos et al., 2018). Perceived teacher control was measured using the four-item Controlling Teacher Scale (CTS—Jang et al., 2009) adapted for Peru (Matos et al., 2016). The autonomy support and controlling teaching styles perceived by the students were assessed considering the music (instrument) performance teaching professor. All the students who answered the questionnaire were referring to professors of these courses. Some of the items are *Header: In class, when the professor teaches me how to play an instrument… “I feel that my professor gives me options and possibilities to choose different ways”* (autonomy support) and “*My teacher puts a lot of pressure on me”* (control). In this study, both questionnaires were analyzed together in a single CFA considering Autonomy Support and Control as latent variables as has been previously done (Matos et al., 2018). CFA obtained satisfactory fit indices (χ^2^**=** 78.79, df = 34, *p* < 0.001, CFI = 0.97, TLI = 0.96, RMSEA = 0.095, SRMR = 0.077). Perceived autonomy support had factor loadings ranging from 0.54 to 0.84, and Cronbach's alpha internal consistency coefficient was 0.82. Perceived teacher control had factor loadings ranging from 0.48 to 0.65 and a Cronbach's alpha of 0.58. Although 0.58 is a low coefficient, low alpha levels (0.50 or higher) have been justified for research purposes (Nunnally, 1967, 1978 cited in Lance et al., 2006), as interpreting alpha requires taking into consideration the number of items that comprise the scale (Taber, 2018).

#### Basic Psychological Needs

To evaluate need satisfaction and frustration we used the 24 item Basic Psychological Need Satisfaction and Frustration Scale (BPNSFS —Chen et al., 2015). This scale was developed in four countries simultaneously (Belgium, USA, China, Peru) showing good psychometric properties in a multigroup analysis and in each country individually. In this sample, the scale obtained adequate fit in a confirmatory factor analysis with need satisfaction and frustration as the two latent variables (χ^2^ = 597.18, df = 251, *p* < 0.001, CFI = 0.97, TLI = 0.97, RMSEA = 0.097, SRMR = 0.084). The need satisfaction scale had adequate reliability (Cronbach's alpha = 0.90) and high factor loadings (ranging from 0.57 to 0.88). The need frustration also obtained adequate reliability (Cronbach's alpha = 0.87) and somewhat lower, yet acceptable factor loadings (ranging from 0.30 to 0.81). Examples of items for each scale are *Header: When I am in this music class…. “I feel I have been doing what really interests me,” “Most of the things I do, feel like ‘I have to”’*.

#### Perfectionism

Perfectionism was measured using the short form of the Revised Almost Perfect Scale (Rice et al., 2014), which uses four items to measure discrepancy (maladaptive perfectionism), and four items to measure standards (adaptive perfectionism). Examples of items for each dimension are “*My highest accomplishment is never good enough to me.” “I have high standards in relation to my musical performance in class.”* This questionnaire has been adapted and translated in prior research in Peru (Herrera et al., 2011). In this sample, the two-factor model fit was appropriate (χ^2^ = 41.56, df = 19, *p* = 0.002, CFI = 0.98, TLI = 0.97, RMSEA = 0.090, SRMR = 0.079). The factor loadings ranged from 0.69 to 0.75 for standards and 0.68 to 0.78 for discrepancy. Reliability was adequate for both subscales: standards (Cronbach's alpha = 0.74) and discrepancy (Cronbach's alpha = 0.79).

#### Flourishing

Flourishing was measured using Diener et al. (2010) eight-item Flourishing Scale, which has been translated and adapted to the Spanish speaking context of Peru (Cassaretto and Martinez, 2017). Two items were not considered because of discriminant validity concerns with basic need satisfaction: these two items overlapped with relatedness satisfaction and competence satisfaction (item 2 “*My social relationships are supportive and rewarding”* and item 5 “*I am competent and capable in the activities that are important to me”*). This six-item version obtained adequate psychometric properties (χ^2^ = 17.84, df = 9, *p* < 0.001, CFI = 0.99, TLI = 0.99, RMSEA = 0.082, SRMR = 0.030. Cronbach's alpha = 0.89. Range of factor loadings = 0.66–0.83).

### Design and Data Analytic Strategy

This is a cross sectional study. In all analyses, R Statistics Software Lavaan package was employed (Rosseel, 2012). We used confirmatory factor analysis (CFA) to generate factor scores which were then used as input for the path models looking at the relationship between variables. This approach has been used before in SDT research, as it preserves factor loading information in the scores, making them a more valid estimation of the latent variable than mean scores (DiStefano et al., 2009; Albuquerque et al., 2017).

We used maximum likelihood estimation with robust standard errors as well as a Satorra-Bentler scaled test statistic (i.e., MLM) which is robust to ordinal data and non-normal distributions (Brown, 2015). We built a path model according to the bright and a dark side of motivation (Haerens et al., 2015) where autonomy support predicts flourishing and adaptive perfectionism (standards), mediated by need satisfaction; while controlling style predicts maladaptive perfectionism (discrepancy), mediated by need frustration. We computed indirect, direct, and total effects to assess the mediational role of need satisfaction and frustration between context variables and outcomes. We allowed our outcomes to be correlated, because prior research has established that adaptive perfectionism is related to well-being (e.g., Rice and Mirzadeh, 2000; McArdle and Duda, 2004).

The fit of path models was evaluated using multiple fit indices: RMSEA, SRMR, CFI, and TLI. According to Hu and Bentler (1999) we aimed for values of CFI higher than 0.95, RSMEA lower than 0.07 and SRMR lower than 0.09.

## Results

### Descriptive and Correlation Analyses

Means, standard deviations and correlations among the studied variables can be seen in Table 1, and several significant correlations were found among the studied variables. From the demographic variables, only sex obtained significant point-biserial correlations with the studied variables. Sex correlated positively with need satisfaction and flourishing while negatively with need frustration.

**Table 1:** Means, standard deviations and correlations between the studied variables.

**Variable**	**1^**a**^**	**2**	**3**	**4**	**5**	**6**	**7**	**8**
1. Sex^a^								
2. Autonomy support	0.14							
3. Control	−0.06	−0.63^***^						
4. Need satisfaction	0.20^*^	0.46^***^	−0.29^***^					
5. Need frustration	−0.19^*^	−0.33^***^	0.24^**^	−0.65^***^				
6. Standard	0.13	0.27^***^	−0.10	0.44^***^	−0.28^***^			
7. Discrepancy	−0.14	−0.15	0.02	−0.37^***^	0.55^***^	0.00		
8. Flourishing	0.18^*^	0.25^**^	−0.11	0.69^***^	−0.47^***^	0.45^***^	−0.36^***^	
Mean	–	5.37	2.93	5.63	2.88	5.73	4.13	5.77
Standard deviation	–	1.05	1.08	0.87	1.02	0.84	1.30	0.93

*Note. N = 149; a = point biserial correlations. Correlations are based on factor scores*.

* *p < 0.05*,

** *p < 0.01*,

*** *p < 0.001*.

As expected, autonomy support was negatively related to control, positively related to need satisfaction, negatively related to need frustration, and positively related to standards and to flourishing. Control in turn, was positively related to need frustration and negatively to need satisfaction. Need satisfaction and frustration were negatively related to each other, and showed significant relations to flourishing, standards and discrepancy, with need satisfaction being related to positive outcomes (and negatively related to negative outcomes). Finally, flourishing was positively correlated to standards, and negatively related to discrepancy.

### Path Analysis

We tested the hypothesized model (Figure 1). To control for the potential effects of sex, models included sex as a predictor of need satisfaction, need frustration and flourishing, since there were significant point biserial correlations between sex in these three variables (see Table 1). However, no significant effects were found for these variables, so they are not presented.

The model obtained optimal fit, which is expected under a model that is almost fully saturated [χ^2^ (7, *N* = 143) = 7.48, *p* = 0.300, CFI = 0.998, TLI = 0.992, RMSEA = 0.021, SRMR = 0.040].

**Figure 2:**
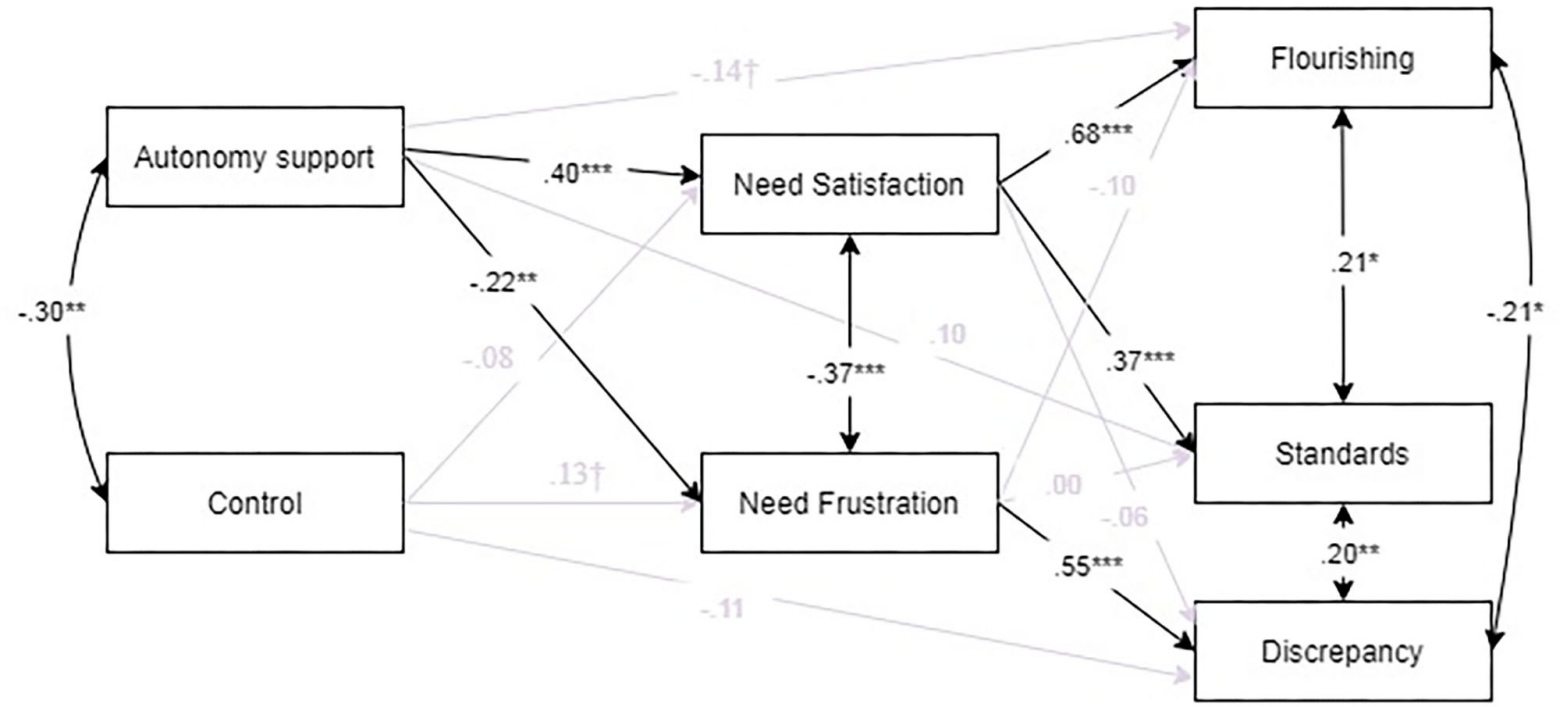
Path model.

Figure 2 shows standardized path coefficients, as well as the direct and indirect effects. The perception of teacher autonomy support, positively predicted need satisfaction and negatively predicted need frustration. In turn, need satisfaction predicted positive outcomes (flourishing and adaptive or high standard perfectionism) while need frustration, positively predicted maladaptive perfectionism (discrepancy).

The model accounted for 48% of the variance in flourishing, 32% of the variance in maladaptive perfectionism (discrepancy) and 17% of the variance of adaptive perfectionism (standards). The mediators, need satisfaction and frustration, have more modest proportions of variance explained (21 and 10%, respectively). Need satisfaction was a significant mediator between autonomy support and flourishing (standardized indirect effect = 0.27, *p* = 0.003) and standards (standardized indirect effect = 0.15, *p* = 0.002).

Following Kenny (2018), we only report proportion of mediation for variables where the absolute standardized total effect is greater than.20. In our sample this is only the case for the mediation between autonomy support, need satisfaction and standards, where 60.10% of the relationship between autonomy support and standards is mediated by need satisfaction.

## Discussion

The aim of the present cross-sectional study was to investigate the role of perceived motivational context, specifically, of perceived autonomy supportive or controlling teaching motivating styles, in predicting adaptive or maladaptive perfectionism and flourishing *via* basic psychological need satisfaction or frustration in University music students. We expected that perceived autonomy supportive teaching style would positively predict flourishing and adaptive perfectionism (i.e., high standards) *via* basic psychological need satisfaction; and, in turn, perceived controlling teaching style would positively predict maladaptive perfectionism (i.e., discrepancy) *via* basic psychological need frustration.

The results of the present study suggest that adaptive and maladaptive forms of perfectionism are related to contextual influences. This is consistent with views in which a person-context interaction shapes perfectionism (Flett et al., 2002; Hibbard and Davies, 2011; Hewitt et al., 2017; Curran and Hill, 2019). Since the model fit was optimal, these findings suggest that motivational contexts in which expectations or criticism from others might be relevant for students' experiences (McArdle and Duda, 2004; Cupido, 2018) may set crucial conditions for need satisfaction and frustration, in turn, prompting adaptive and maladaptive perfectionism, and flourishing.

Focusing particularly on a highly demanding environment such as University music education programs, it is possible to argue that this type of context can play a role in students' motivational dynamics and its outcomes. In this respect, our findings show, firstly, that a perceived autonomy supportive teaching motivational style is a positive predictor of basic psychological need satisfaction, adaptive perfectionism (standard), and flourishing, in line with theoretical postulates (Ryan and Deci, 2017; Nordin-Bates and Kuylser, 2020).

On the other hand, as expected, need frustration did indeed predict maladaptive perfectionism (discrepancy) positively; but breaking away from theoretical expectations, our results did not support that controlling teaching motivational style would predict need frustration. Instead, a path that had not been expected appeared in the model, namely, autonomy support negatively predicted need frustration. However, these unexpected findings are consistent with the theoretical framework of SDT.

To put the findings into perspective, it is important to note that our research is aligned with SDT's notions of bright and dark sides of motivational dynamics. According to relevant research within SDT (Haerens et al., 2015), the first route, or “bright” side, implies that perceived autonomy supportive teaching style (context) is expected to predict autonomous motivation positively *via* basic psychological need satisfaction, ultimately, leading to well-being (Vansteenkiste and Ryan, 2013); and that, a “dark” side would go from perceived controlling teaching style toward controlled motivation, amotivation, and oppositional defiance, via basic psychological need frustration (Haerens et al., 2015). Considering these two paths, different studies examined what has been named as the bright and dark sides of motivation (Rodrigues et al., 2020).

As described above, these notions have been partially supported by our results, insofar as the found effects were as expected on the bright side, but not totally on the dark side. In this sense, our findings suggest that indeed context matters, given that—at least on the bright side—the motivating style of the teacher predicted the outcomes of well-being such as adaptive perfectionism and flourishing, via the positive effects that they can have on basic psychological need satisfaction. Notwithstanding—on the dark side—our findings did not fully support the postulated motivational dynamics: basic psychological need frustration positively predicted maladaptive perfectionism (discrepancy) but, in contrast with our expectations, controlling teaching style did not predict need frustration positively, and in turn need frustration was predicted negatively by autonomy supportive teaching motivating style, in this way accounting for the occurrence of a crossed path not considered in the hypothetical model of independent bright and dark sides of motivation. However, perceived autonomy supportive motivating style negatively predicted the frustration of basic psychological needs. This path signals that, in this sample of music students, psychological need frustration may not necessarily be predicted by higher perceived controlling motivating style, but instead by different levels of autonomy supportive style. Further studies in samples of music students will be required to assess if this relation between autonomy support and need frustration finds replication, and also to consider if -in such a case- this association would be specific to University music students' motivational dynamics. Another reason why the theoretically expected relationship between controlling teaching style and need frustration was not observed, could be measurement error; as controlling teaching style was measured using a four-item scale which obtained a low reliability coefficient. As such, it is possible that measurement error might have suppressed the relationship between control teaching style and need frustration which exists according to theory.

The present findings allow us to articulate both the adaptive (standard) and maladaptive (discrepancy) dimensions of perfectionism with, respectively, the bright and dark sides of motivational dynamics postulated by SDT (e.g., Haerens et al., 2015). In this respect, the standard perfectionism dimension appears to be located within a network of bright side variables aligned with well-being (e.g., need satisfaction and flourishing); whereas, the discrepancy dimension of perfectionism would have to be considered as a part of a dark side variable network, including need frustration and potentially other ill-being variables, such as maladaptive perfectionism or discrepancy. We suggest that future studies could also ponder the role of adaptive or standard perfectionism for well-being, including the analyses of its potential interactions with performance in the occurrence of flourishing, or its potential mediating effects between contextual aspects (e.g., teaching motivational styles) and well-being.

Lastly, this study sheds light on motivational processes leading to well-being through the facilitation of the conditions that enable people's growth and flourishing. The present findings are especially relevant for understanding the experiences of University music students who must cope with contextual pressure to perform at a very high level, which, as mentioned above, could lead to either adaptive or maladaptive outcomes. Future studies could employ longitudinal designs to analyze the stability and change of perfectionism in music students. It would be especially interesting to analyze if changes in perceived teacher motivating style longitudinally predict change in perfectionism.

It is important to mention at this point that there are some limitations to this research. Firstly, a higher number of participants would have been desirable, in order to gain a greater power to test the relationships derived from the model. However, the population of interest in Peru is both small and difficult to access. Secondly, given the cross-sectional design of the present study, relationships among variables could be described at one specific moment only, rendering impossible the prediction of long term contextual effects on adaptive and maladaptive perfectionism.

Considering that the main aim of research within human sciences is to uncover the psychological processes leading to improving human condition (Ryan and Deci, 2000), it is indispensable to acknowledge that, as humans, we have both the potential for growth, integration, and well-being; but also, the vulnerability to defensiveness, aggression, and ill-being (Vansteenkiste and Ryan, 2013). Accordingly, the present contribution opens new ways of analyzing what is psychologically happening in a music class. Universities could profit from these initial findings in the field of Peruvian higher music education to be able to contribute to the description, explanation, and prediction of human behavior, as well as to apply these to the promotion of positive human experience in higher music education (Herrera et al., 2018).

It is possible to conclude that motivational dynamics in University music students are articulated as two competing influences: one, in which contextual basic psychological need support leads to need satisfaction, well-being and adaptive perfectionism; and another, in which basic psychological need thwarting leads to need frustration and maladaptive perfectionism. This way of enhancing motivation and its positive outcomes through contextual support provides clear arguments for researchers and policy makers in the field of higher music education, to continue deepening the knowledge about how to design and implement pedagogical processes leading to the development of inner motivational resources. Teachers can be trained to provide autonomy support to benefit students' motivational processes (Cheon et al., 2020). Nonetheless, on the dark side of motivation, specifically in University music students, further research will be necessary to more precisely describe the relationships between contextual and personal variables.

## Data Availability Statement

The raw data supporting the conclusions of this article will be shared with interested scholars who request them from the corresponding author.

## Ethics Statement

The study, involving human participants, was reviewed and approved by the Research Ethics Committee of the Pontifical Catholic University of Peru. Participants were required to provide informed consent/assent in written form.

## Author Contributions

DH principal investigator and in charge of preparing, coordinating, and discussing the results and final version of the manuscript. LM main colaborator in the theoretical and empirical section of the article. RG collaborator in theoretical and methodological aspects. BL main colaborator in statistical analysis and results reporting. RV main colaborator in the theoretical discussion of the manuscript framework, data collection, and practical implications. All authors contributed to the article and approved the submitted version.

## Conflict of Interest

The authors declare that the research was conducted in the absence of any commercial or financial relationships that could be construed as a potential conflict of interest.
